# Uniaxial Compressive Stress–Strain Model of Jujube Nucleus Concrete following Exposure to Elevated Temperatures

**DOI:** 10.3390/ma16031037

**Published:** 2023-01-24

**Authors:** Jieqi Li, Mingming Jia, Shan Gao, Jian Yuan

**Affiliations:** 1 School of Civil Engineering, Xijing University, Xi’an 710123, China; 2 Key Lab of Structures Dynamic Behavior and Control of the Ministry of Education, Harbin Institute of Technology, Harbin 150090, China; 3 Academy of Combat Support, Rocket Force University of Engineering, Xi’an 710025, China

**Keywords:** agricultural waste, coarse aggregate, elevated temperature, modified concrete, stress–strain relationships

## Abstract

Aiming to provide a solution for natural resource consumption and agricultural waste pollution, jujube nucleus is utilized as a substitute for coarse aggregate in the preparation of lightweight aggregate concrete. The effect of the jujube nucleus (JN) replacement ratio and the elevated temperature on the uniaxial compressive stress–strain curves of jujube nucleus concrete (JNC) are experimentally studied. The results show that the failure of the JNC prisms became more serious with the increase in the JN replacement ratio. The linear proportion in ascending branch and the descending slope of the stress–strain curves for JNC increased gradually with the increase in the JN replacement ratio and elevated temperature, which is probably owing to the higher porosity and lower stiffness of the jujube nucleus, compared to natural aggregate. Moreover, as the JN replacement ratio and the elevated temperature increase, the peak stress and elastic modulus in the stress–strain curves of JNC decrease gradually, whilst an increase in the peak strain shows up, which is possibly due to the growth of hydrate calcium silicate and calcium hydroxide hampered by sucrose molecules. Based on the test results, a series of theoretical formulas are proposed to predict the compressive performance of JNC. A material constitutive model is developed for describing the stress–strain relationship of JNC by considering the JN replacement ratio and elevated temperature.

## 1. Introduction

Statistics show that, over the past few decades, the annual consumption of concrete for the construction industry has been up to 8 billion tons globally [1], of which China accounts for approximately 25% [2]. Accordingly, natural aggregates considered as a non-renewable resource tend to deplete as a result of over-exploitation of minerals [3]. It can be seen that gravel and sand is mined beyond 1.1 to 1.4 billion m^3^ in China each year [4], and they make up 70% of concrete production [5]. In addition, large amounts of waste materials are dumped and burned as a result of rapid urbanization, industrialization, and population growth, which is a severe burden on waste management and environmental pollution [6]. As a result, the feasible study of using agricultural by-products as a substitute for coarse aggregate in concrete production has been proposed for alleviating the pressure from ever-increasing natural resource consumption and agricultural waste pollution [7].

In China, jujube nucleus, considered as one of the major agricultural by-products, would be discharged by more than 3 billion tons every year [8]. How to utilize these waste jujube nuclei has become a serious topic in the agricultural industry. Although Xie et al. [9] and Tabrez et al. [10] indicated that jujube nuclei waste can be carbonized to prepare the activated carbon adsorbent in pursuit of adsorbing pigments and their impurities in industrial wastewater, whilst the carbonization process for jujube nuclei has high energy consumption. In contrast, the jujube nucleus has the characteristic of low density and a reasonable gradation feature, which offers the possibility to replace natural coarse aggregates to prepare agricultural waste concrete. Jujube nucleus as coarse aggregate in structural concrete not only avoids the secondary treatment of the waste, but also reduces the self-weight of structural concrete [11].

The mechanical properties of agricultural waste concrete have been investigated for decades. However, study on the stress–strain relationship of agricultural waste concrete under uniaxial compressive loading is rarely involved. The effect of coconut shell (CS) as an alternative coarse aggregate for concrete materials was studied by Tomas and Ganiron [12]. The results showed that as the CS replacement ratio increases, the compressive strength of coconut shell concrete decreases gradually. Gunasekaran et al. [13] pointed out that the utilization of coconut shells as coarse aggregate in agricultural waste concrete reduces the bond between cement and coarse aggregate, which causes the compressive strength degradation of coconut shell concrete. Alengaram et al. [14] investigated the application of palm shell (OPS) as a substitute for coarse aggregate in concrete production. The results showed that palm shell concrete has comparable mechanical properties with ordinary concrete when the palm shell replacement ratio is low. However, the compressive strength and splitting tensile strength of palm shell concrete decrease rapidly as the palm shell replacement ratio increases. Khan et al. [15] found that palm shells are less bonded to cement compared with natural aggregate, which is the reason for the strength degradation of palm shell concrete. Muthusamy et al. [16] concluded that the mechanical properties of rubber concrete gradually decrease as the rubber replacement ratio increases, because the rubber and the cement paste does not cover the aggregates uniformly. Memon et al. [17] investigated the compressive performance of agricultural waste concrete using corncob ash as a substitute for fine aggregate. The results showed that the compressive strength degradation of the concrete may be related to the high initial free water content and the inherently weaker mechanical properties of corncob ash. 

The fire resistance of concrete materials is one of the indices to evaluate the durability of concrete in engineering structures [18], which features structural design. Therefore, a number of studies on the effect of high temperature on the mechanical properties of agricultural waste concrete have been conducted [19]. However, the effect of high temperature on the stress–strain relationship of agricultural waste concrete is barely involved. Mo et al. [20] investigated the compressive strength of palm shell concrete after elevated temperatures. The results show that after exposure to elevated temperature within the range of 200 °C to 400 °C, the loss in compressive strength of palm shell concrete is about 30% to 55%, respectively. The compressive strength of coconut concrete after elevated temperatures was experimentally studied by Gunasekaran et al. [21]. The results show that, according to the exposure duration, the compressive strength attenuation of coconut concrete is approximately 25–50% and 55–75% at temperatures of 200 °C and 400 °C, respectively. 

**Figure 1:**
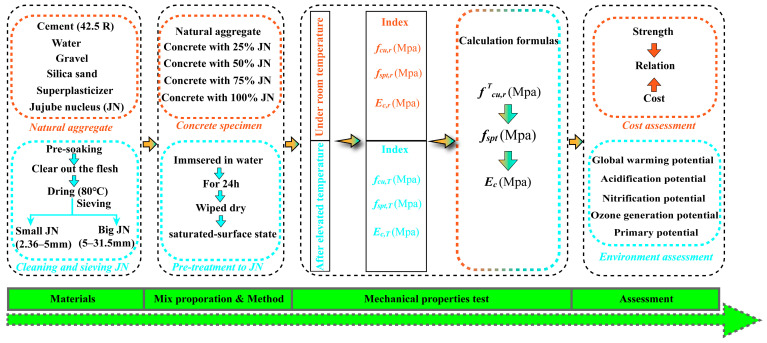
Research flow chart of the experiment.

Based on the above-mentioned review, a lack of data on the stress–strain relationship of agricultural waste concrete under uniaxial compressive behavior is indicated. Therefore, the jujube nucleus is utilized herein as a coarse aggregate in the preparation of agricultural waste concrete, as shown in Figure 1. The failure phenomena, uniaxial compressive performance, and the stress–strain model of jujube nucleus concrete (JNC) under the effect of the jujube nucleus (JN) replacement ratio and the elevated temperature are investigated experimentally and discussed in detail. The prediction formulas for the stress–strain constitutive model of JNC are proposed by considering the JN replacement ratio and the elevated temperature. 

## 2. Materials and Methods

### 2.1. Materials

#### 2.1.1. Natural Aggregate, Cement, and Water

The concrete mixture was prepared by using natural silica sand and natural limestone gravel sourced from local suppliers, Xi’an, China. Following China’s standard JGJ 52-2006 [22], the physical properties of the natural coarse aggregates were tested as shown in Table 1; 42.5R ordinary Portland cement and tap water were used to act as an adhesive for the concrete mixture, while a superplasticizer with 3% of the cement weight was used to reinforce the workability of the mixture.

#### 2.1.2. Jujube Nucleus

**Figure 2:**
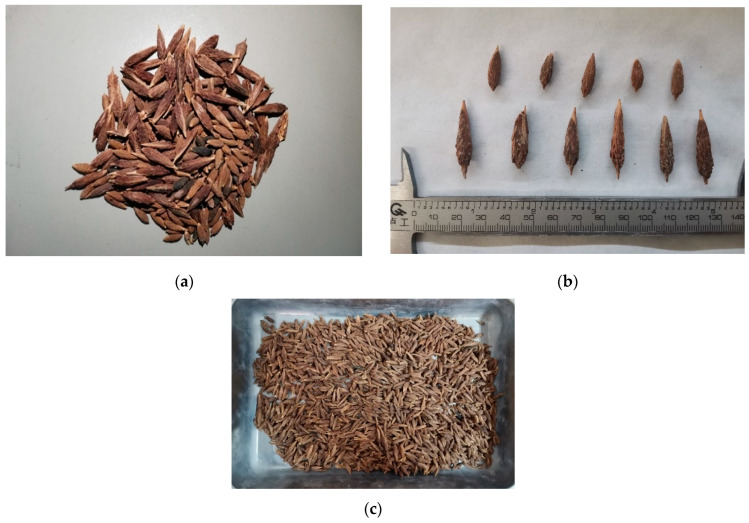
Jujube nuclei used in the test. (**a**) Jujube nuclei after pretreatment. (**b**) The particle size of jujube nuclei. (**c**) Jujube nuclei used in the specimen preparation.

The jujube nucleus used herein as a substitute for natural coarse aggregate was collected from a fruit processing facility located in Shaanxi province, China. The surface of the jujube nucleus was generally enveloped by large amounts of unripped flesh that was abundant in saccharides such as glucose, fructose, sucrose, oligosaccharide, arabinose, and galactose [10]. Saccharides that dissolved in the water during the concrete preparation process could interfere with the hydration of the cement, which has a noticeable influence on the mechanical properties of concrete [23,24]. Referring to previous studies [25], these jujube nuclei were first soaked in water for 24 h, and then rinsed by a high-pressure water gun in order to remove these fleshes. These jujube nuclei were then baked in a high-temperature oven for 2 h to make their surface completely dry (the elevated temperature was 80 °C). As shown in Figure 2a, the surface of these treated jujube nuclei is rough without the residual flesh.

Following the Chinese standard GB/T 17431.2-2010 [26], these jujube nuclei were sieved for the preparation of lightweight aggregate concrete. The particle sizes of the sieved jujube nuclei were in the range of 2.36–31.5 mm, of which 5–31.5 mm was large jujube nucleus, and 2.36–5 mm was small jujube nucleus, as seen in Figure 2b.

The physical properties of the jujube nucleus were tested, as shown in Table 2. Compared with the natural coarse aggregates, the jujube nucleus showed higher water absorption, but lower apparent density, implying porous structural characteristics. In order to remove the influence of water absorption on test results [27], these jujube nuclei were immersed in water for 24 h prior to specimen preparation to make them absorb water adequately, and they were then wiped to attain a saturated surface dry state for specimen preparation, as shown in Figure 2c.

### 2.2. Mixture Design and Specimen Preparation

#### 2.2.1. Mixture Proportions of Concrete

For the ordinary concrete without jujube nucleus, the mixture proportion was designed according to China’s JGJ55-2011 standard [28]. Under the same mixture proportion design, concrete with jujube nucleus was prepared using the waste jujube nucleus as a substitute for natural coarse aggregate with the replacement ratio of 25%, 50%, 75%, and 100%, respectively (by volume) in the preparation of jujube nucleus concrete. The mixture proportions of ordinary concrete and jujube nucleus concrete are listed in Table 3.

#### 2.2.2. Preparation of Specimens

Prisms of 150 mm × 150 mm × 300 mm were prepared for testing the uniaxial compressive strength, elastic modulus, peak strain, and the whole stress–strain curves of JNC (three parallel specimens for each concrete mixture proportion). Specimen identification, as listed in Table 4, begins with specimen type followed by the target elevated temperature and the JN replacement rate.

### 2.3. Testing Procedure

#### 2.3.1. Mechanical Properties Test under Room Temperature

**Figure 3:**
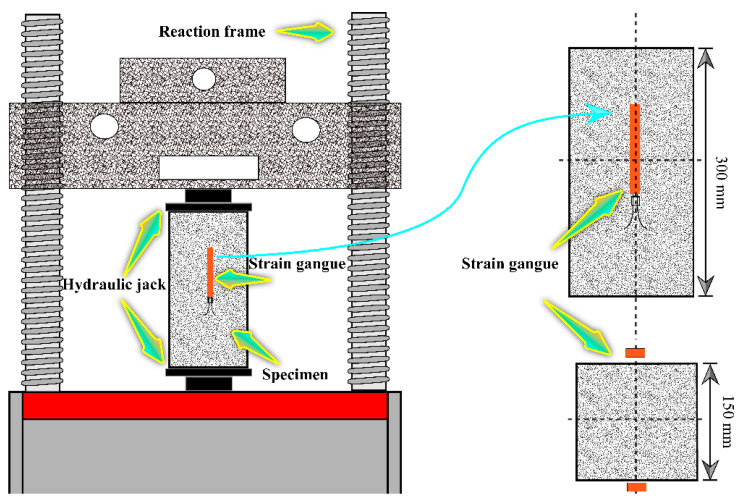
Test machine and arrangement method.

The uniaxial compression test of JNC was performed by a hydraulic compression machine with a maximum test load of 2000 kN. Specifically, a force control method was used to impose compression on the sample at a speed of 1 kN/s. Once the load reached 15 kN, the force-controlled method was used via the displacement-controlled method at a rate of 0.025 mm/s until the specimens failed. Two strain gauges were arranged on each side of the specimen to monitor the longitudinal strain, as shown in Figure 3.

#### 2.3.2. Heating Regime

**Figure 4:**
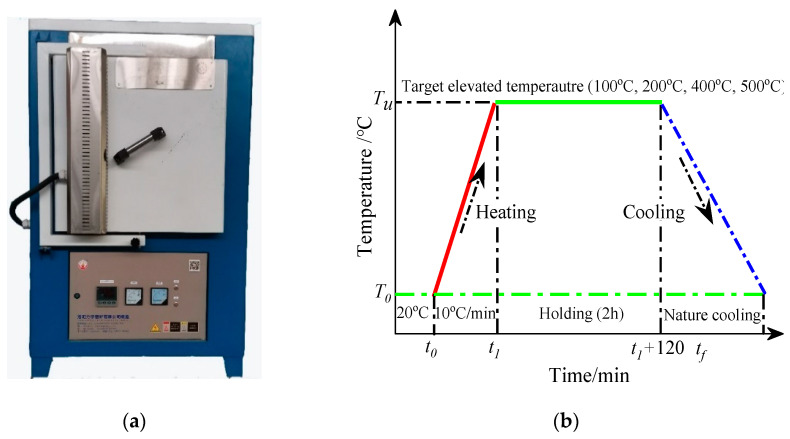
High temperature loading procedure. (**a**) High temperature furnace. (**b**) Heating, holding, and cooling process.

The high temperature furnace in which the concrete specimens were preheated is illustrated in Figure 4a. The maximum output of the high temperature furnace was 18 kW. The dimensions of the chamber were 0.5 m × 0.5 m × 0.5 m. 

Prior to the uniaxial compressive test, the concrete specimens were heated from room temperature to a specific target temperature (100 °C, 200 °C, 400 °C, and 500 °C, respectively) at a rate of 10 °C/min. Mohamedhai et al. [29] pointed out that the influence of high temperature on the mechanical properties of concrete materials occurs primarily in the first couple of hours after exposure to temperature. For this reason, all concrete specimens exposed to high temperature were held in the high temperature furnace for 2 h after reaching the target temperature. The concrete specimens were then cooled down to room temperature by nature-cooling in the furnace. The heating, holding, and cooling temperature regime in this test is illustrated in Figure 4b.

## 3. Results and Discussions

### 3.1. Failure Pattern

**Figure 5:**
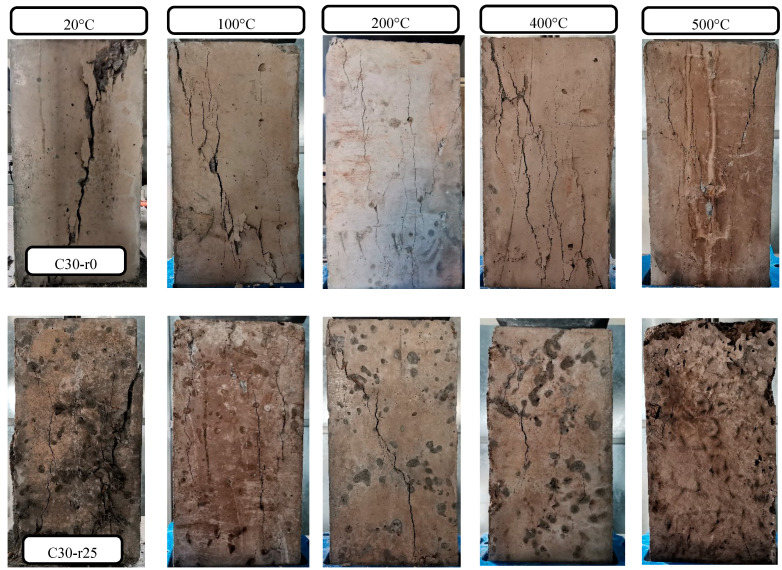

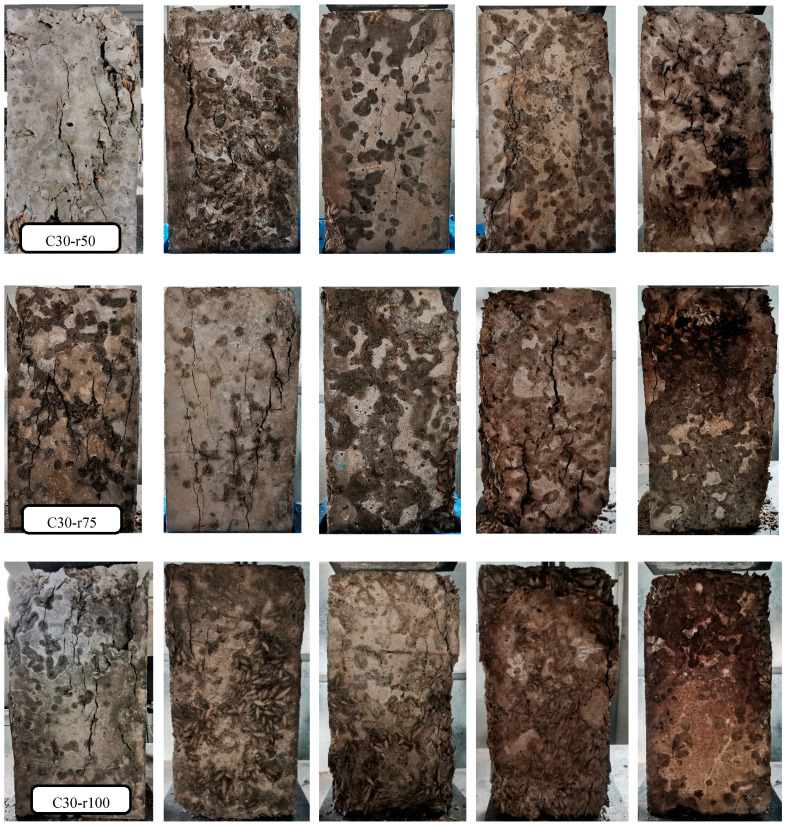
Failure phenomenon of the uniaxial compressive strength test.

The failure phenomena of all the specimens tested under the uniaxial compressive loading for JNC are shown in Figure 5.

For ordinary concrete, namely without jujube nucleus, shear failure was observed in all the specimens, and a main diagonal crack occurred as a result of uniaxial compression. As the elevated temperature increased, more vertical cracks appeared in all the specimens besides the diagonal crack. Under heating, the evaporation of free water and the decomposition of hydrate calcium silicate (C-S-H) and calcium hydroxide resulted in the growing number of pores in the concrete. Thus, the crack propagation of the concrete after elevated temperature under the uniaxial compressive loading only needed to dissipate a little energy, compared to that of ordinary concrete without heating [30]. 

For jujube nucleus concrete (JNC), the number and width of diagonal and vertical cracks increase with the increase in the jujube nucleus replacement ratio and elevated temperature, which is mainly due to the low stiffness and high porosity of jujube nucleus, which may result in the crack propagation pattern being transferred from the damaged interfacial transition zone to aggregate penetrated under the uniaxial compressive loading, reflecting the weaker energy dissipation capacity of JNC [31]. However, the number of diagonal and vertical cracks for JNC decreased gradually with the increase in the elevated temperature, which is probably because the jujube nucleus was burned out under the effect of the elevated temperature, causing the generation of a large pore structure. It was noteworthy that when the jujube nucleus replacement ratio exceeded 25%, the JNC was prone to being squashed, which is due to the ineffectiveness of cementitious materials under high JN replacement ratio [32].

### 3.2. Uniaxial Compressive Stress–strain Curves

#### 3.2.1. Effects of the Elevated Temperature on Stress Strain Curves for JNC

**Figure 6:**
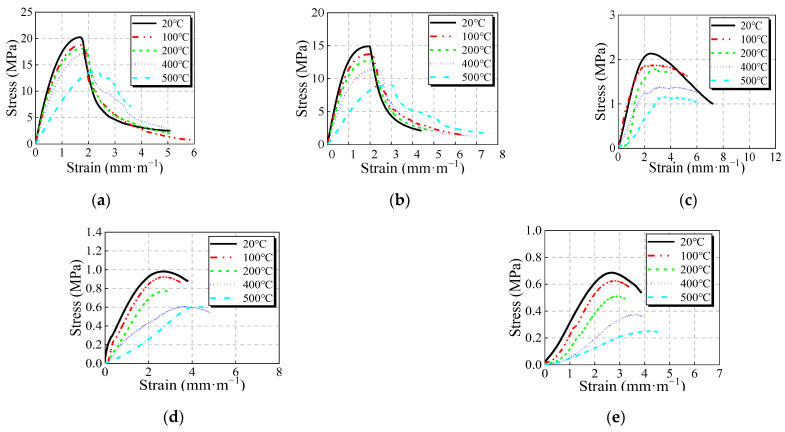
Stress–strain curves for JNC under the same replacement ratio after experiencing the various elevated temperatures. (**a**) *r* = 0%. (**b**) *r* = 25%. (**c**) *r* = 50%. (**d**) *r* = 75%. (**e**) *r* = 100%.

The whole stress–strain curves of JNC under the same JN replacement ratio after experiencing various elevated temperatures is presented in Figure 6. 

**Figure 7:**
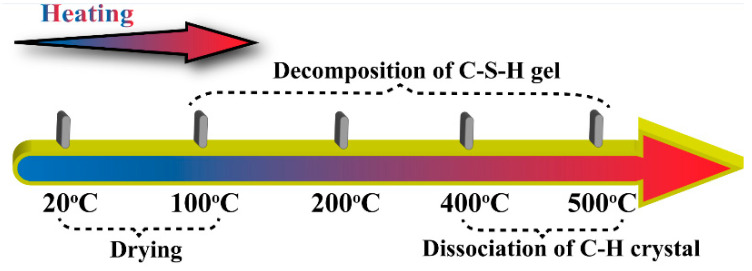
Effect of the elevated temperature on concrete.

It can be seen that as the elevated temperature increases, the peak stress and peak strain of JNC decrease gradually. Additionally, the slope of the stress–strain curves, for both the ascending and descending branches, decreases with the rise in the elevated temperature. It is noted that when the elevated temperature exceeds 400 °C, the peak stress and peak strain of ordinary concrete show a larger reduction than those of JNC. This may be because when the elevated temperature exceeds 400 °C, the Ca (OH)_2_ crystal, having a larger volume in the concrete, begins dissociation, which causes cement paste dehydration [33], as shown in Figure 7. At the same time, the most noticeable thermal expansion promotes crack propagation. The difference is that during the heating procedure, the porosity and rough texture of jujube nucleus may result in less damage to the concrete.

#### 3.2.2. Effects of the Replacement Ratio on Stress–Strain Curves for JNC

**Figure 8:**
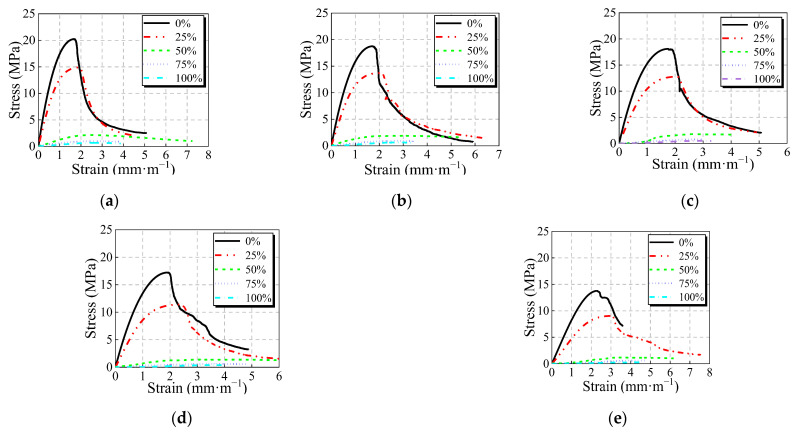
Stress–strain curves with the various jujube nucleus replacement ratio after elevated temperatures. (**a**) *T* = 20 °C. (**b**) *T* = 100 °C. (**c**) *T* = 200 °C. (**d**) *T* = 400 °C. (**e**) *T* = 500 °C.

The whole stress–strain curves of JNC under the same elevated temperature with the various JN replacement ratio are presented in Figure 8.

**Figure 9:**
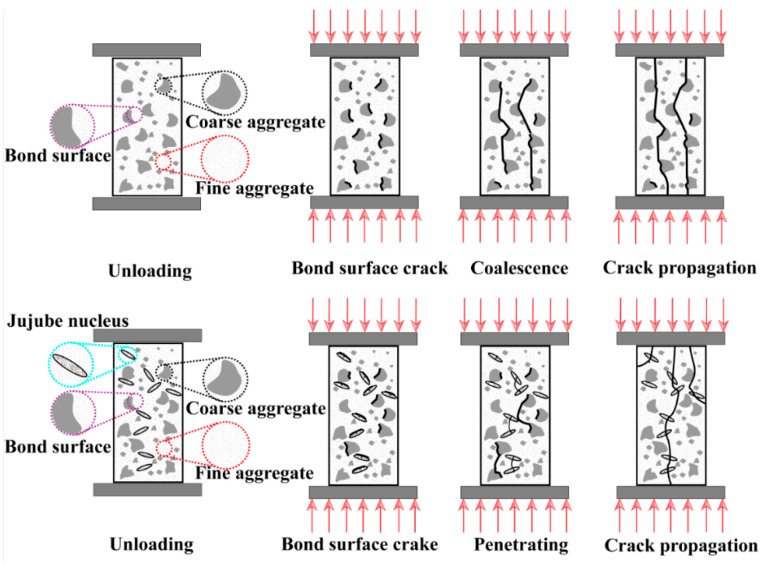
The fracture mechanism of jujube nucleus concrete.

It can be seen from Figure 8 that as the JN replacement ratio increases, the peak stress and ascending branch slope of the mean curves gradually decrease whilst the peak strain and descending branch slope of the mean curves increase relatively under various elevated temperatures. Furthermore, the linear proportion of the curves in the ascending branch increases with the rise in the JN replacement ratio. Fan et al. [31] pointed out that on account of having lower elastic modulus compared to natural aggregate, the lightweight aggregate in concrete is inclined to fracture. For ordinary concrete (without jujube nucleus), the compressive damage is the destruction of the bonding surface of natural aggregate and cement mortar, namely the damaged interfacial transition zone, which proceeds in three stages: bond surface cracks, coalescence, and crack propagation. For lightweight aggregate concrete, the compressive damage is the penetrating of lightweight aggregates, which proceeds in three stages: bond surface cracks, penetrating of lightweight aggregate, and crack propagation, as shown in Figure 9.

### 3.3. Stress Strain Characteristic

After a 28-day curing process, the stress–strain characteristics with various jujube nucleus (JN) replacement ratios after experiencing various elevated temperatures were derived from the stress–strain curves of JNC, including the peak stress, peak strain, and elastic modulus, as tabulated in Table 5. 

#### 3.3.1. Peak Stress of JNC

As shown in Figure 9, each point datum presents the measured average value of the peak stress and its relative value ($f_{c,r}^{T}/f_{c,r}^{20}$ and $f_{c,r}^{T}/f_{c,0}^{T}$) for JNC under the effect of the JN replacement ratio and the elevated temperature. $f_{c,r}^{T}/f_{c,r}^{20}$ is the ratio of the peak stress after experiencing various elevated temperatures to the peak stress without heating under the same JN replacement ratio. $f_{c,r}^{T}/f_{c,0}^{T}$ is the ratio of the peak stress with various JN replacement ratios to the peak stress without replacement ratio under the same elevated temperature.

**Figure 10:**
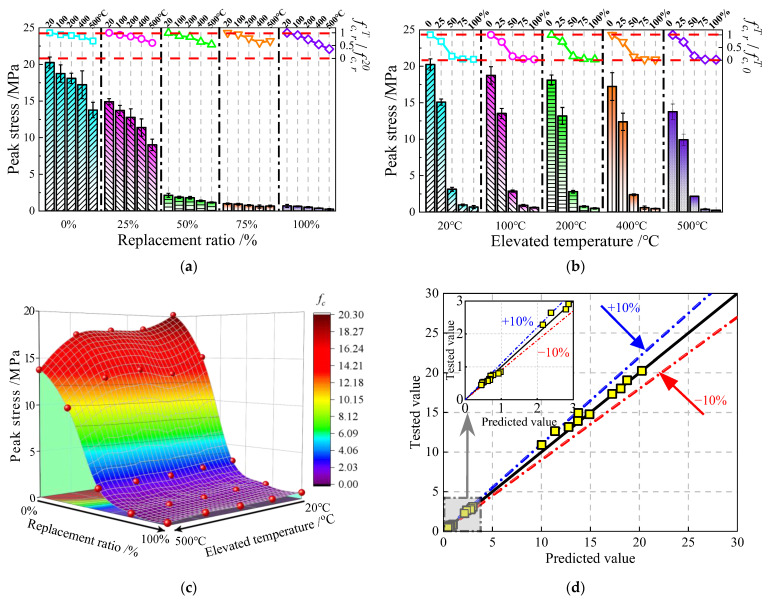
Peak stress of JNC. (**a**) Effect elevated temperature on *f*_c_. (**b**) Effect of replacement ratio on *f*_c_. (**c**) Surface of *f*_c_ under the effect of replacement ratios and elevated temperatures. (**d**) Comparison of tested values and predicted values.

It can be seen from Figure 10a that as the elevated temperature increases, the peak stress of JNC degrades gradually under various JN replacement ratios. The difference in the peak stress between two adjacent temperatures becomes larger when the elevated temperature exceeds 400 °C, which is due to the dissociation of C-H crystal [30]. It can be seen from Figure 10b that the increase in the JN replacement ratio would increase the peak stress of JNC under the various elevated temperatures. The difference in the peak stress between the two adjacent JN replacement ratios becomes larger when the JN replacement ratio exceeds 25%, which is attributed to the rise in sucrose content [32]. Figure 10c shows the peak stress deterioration under the effect of the JN replacement ratio and the elevated temperature. It can be seen that the surface of the peak stress slips down precipitously at the bottom with the rise in the JN replacement ratio and the elevated temperature. 

In order to explicitly express the relationship between the elevated temperature, the JN replacement ratio, and the peak stress, a calculation formula is proposed based on the test results through nonlinear fitting, as given in Equations (1)–(3). Figure 10d shows the comparison of the tested value and the predicted value by using Equations (1)–(3). It can be seen that the deviation of the proposed calculation formula is basically within ±10%.

(1) $$f_{c,r}^{T}=\eta_{c}^{}\lambda_{c}^{}f_{c,0}^{20}$$

(2) $$\eta_{c}^{}=\left\{ \begin{array}{cc} 1-0.41(\frac{T-20}{500})+0.29{\left(\frac{T-20}{500}\right)}^{2}\; & 20{}^{\circ}C\leq T\leq 400{}^{\circ}C\\ 3.31-2.68(\frac{T-20}{500})\; & 400{}^{\circ}C<T\leq 500{}^{\circ}C \end{array}\right.$$

(3) $$\lambda_{c}=\left\{ \begin{array}{cc} 1-1.08r & 0\leq r\leq 25\%\\ 0.67-1.43r+0.79r^{2} & 25\%<r\leq 100\% \end{array}\right.$$

where *η*_c_ is the reduction factor of the relative value ($f_{c,r}^{T}/f_{c,r}^{20}$), *λ*_c_ is the reduction factor of the relative value ($f_{c,r}^{T}/f_{c,0}^{T}$), $f_{c,r}^{T}$ is the peak stress of JNC under the effect of the JN replacement ratio and the elevated temperature, and $f_{c,0}^{20}$ is the peak stress of JNC without jujube nucleus and heating. 

#### 3.3.2. Elastic Modules of JNC

**Figure 11:**
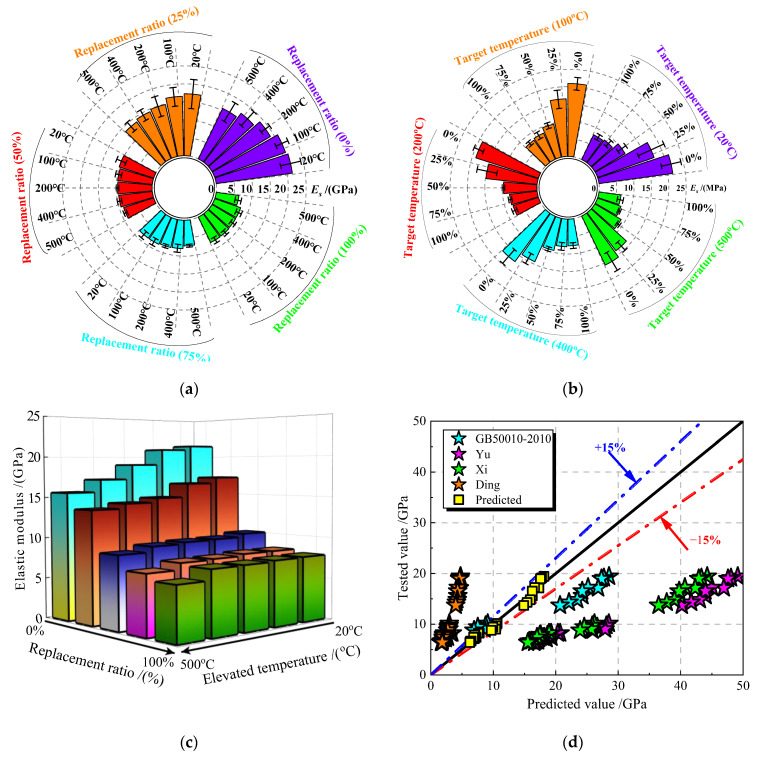
Elastic modulus test of JNC. (**a**) Effect of elevated temperature on *E*_c_. (**b**) Effect of replacement ratio on *E*_c_. (**c**) Variation of *E*_c_ under the effect of replacement ratios and elevated temperatures. (**d**) Comparison of the tested values and the predicted values [34,35,36,37].

The elastic modulus of JNC is taken as the secant modulus from the origin to 40% of the peak stress. Figure 11 presents the measured average value of elastic modulus for JNC under the effect of the jujube nucleus (JN) replacement ratio and the elevated temperature.

Analogous to the peak stress of JNC, the elastic modulus of JNC decrease gradually with the rise in the JN replacement ratio and the elevated temperature, as shown in Figure 11a–c. To be specific, when the elevated temperature exceeds 400 °C, the elastic modulus of JNC within the various replacement ratios (0% (control group), 25%, 50%, 75%, and 100%) is reduced by about 20%, 29%, 55%, 63%, and 67%, respectively, whilst the elastic modulus of JNC after experiencing various elevated temperature (20 °C (control group), 100 °C, 200 °C, 400 °C, and 500 °C) is decreased by approximately 11%, 15%, 24%, 26%, and 29%, respectively, when the JN replacement ratio reaches 25%. 

To predict the elastic modulus of concrete materials, Equations (4) and (5) are recommended by GB/T 50010-2010 [34] and Yu [35] (a = 20805, b = 0.29), respectively, to predict the elastic modulus of ordinary concrete, whilst Equation (6) is put forward by Xi [36] (a = 1.60, b = 1.347, and c = 0.275) and Ding [37] (a = 2.09, b = 1, c = 0.25) to calculate the elastic modulus of lightweight aggregate concrete. Figure 11d shows the comparison of the tested values and the predicted values. It can be seen that Ding is prone to overestimate the elastic modulus of JNC, while the formulas from Yu, Xi, and GB 50010-2010 are inclined to underestimate the elastic modulus of JNC. Therefore, Equation (7) is proposed based on the test results to predict the elastic modulus of JNC, namely, a = 2.38, b = 1.16, and c = 0.28. The discrepancy of the proposed constants is basically within ± 15%, as shown in Figure 11d.

(4) $$E_{c}^{}=\frac{10^{5}\;}{2.2+\frac{26.37}{f_{c}}}$$

(5) $$E_{c}^{}=a\;f_{c}^{b}\;$$

(6) $$E_{c}^{}=a\;\rho^{b}f_{c}^{c}$$

(7) $$E_{c}^{}=2.38\;\rho^{1.16}f_{c}^{0.28}\;$$

where, a and b are constants, and *ρ* is the density of the jujube nucleus.

#### 3.3.3. Peak Strain of JNC

**Figure 12:**
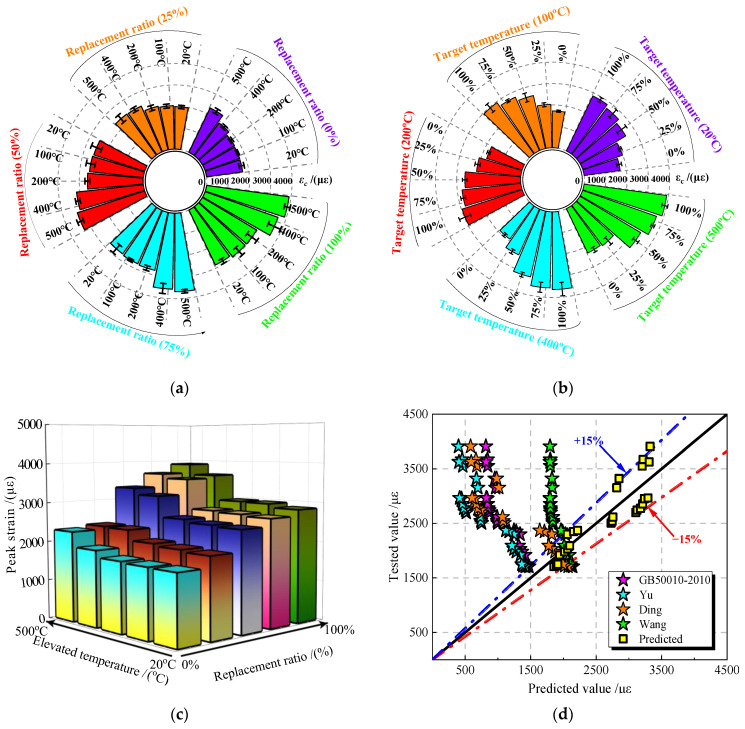
Peak strain of JNC. (**a**) Effect of elevated temperature on *ε*_c_. (**b**) Effect of replacement ratio on *ε*_c_. (**c**) Variation of *ε*_c_ under the effect of replacement ratios and elevated temperatures. (**d**) Comparison of the tested values and the predicted values [34,35,37,38].

The peak strain is the strain at maximum uniaxial compressive strength. Figure 12 shows the measured average value of JNC under the effect of the JN replacement ratio and the elevated temperature.

It can be seen from Figure 12a that the increase in the elevated temperature would increase the peak strain of JNC under various JN replacement ratios (0% (control group), 25%, 50%, 75%, and 100%). The difference in peak strain between the two adjacent temperatures becomes larger when the elevated temperature exceeds 400 °C, increased by about 35%, 39%, 94%, 115%, and 129%, respectively. As shown in Figure 12b, the rise in the JN replacement ratio increase similarly to the peak strain of JNC under the various elevated temperature (20 °C (control group), 100 °C, 200 °C, 400 °C, and 500 °C). The difference in the peak strain between two adjacent replacement ratios becomes larger when the JN replacement ratio reaches 25%, increased by approximately 15%, 19%, 22%, 38%, and 39%, respectively. Figure 12c shows the peak strain of JNC under the effect of the JN replacement ratio and the elevated temperature. It can be seen that the rise in the JN replacement ratio and the elevated temperature increases the peak strain of JNC. It is worth noting that the JN replacement ratio also has a notable effect on the peak strain of JNC, which is analogous to the reason for the peak stress deterioration for JNC. 

To predict the peak strain of concrete materials, Equation (8) is recommended by Yu [35] (a = 520, b = 0.33) and Ding [37] (a = 760, b = 0.33), while Equation (9) is used by GB50010-2010 [34] (a = 172, b = 0.5, and c = 700) and Wang [38] (a = 17.48, b = 1, and c = 1788). Figure 12d shows the comparison of the tested values and the predicted values. It can be seen that all the formulas proposed tend to overestimate the peak strain of JNC. Based on the test results, Equation (10) is proposed to predict the peak strain of JNC, namely, a = −1510.82, b = 0.2, and c = 4611.82. The error of the proposed constants is basically within ±15%, as shown in Figure 12d.

(8) $$\varepsilon_{c}^{}=a\;f_{c}^{b}\;$$

(9) $$\varepsilon_{c}^{}=a\;f_{c}^{b}+c$$

(10) $$\varepsilon_{c}^{}=(4611.82-1510.82f_{c}^{0.2})\times 10^{-6}$$

where a and b are constants, and *ρ* is the density of the jujube nucleus.

## 4. Constitutive Model of JNC 

### 4.1. Suggested Stress–Strain Model for JNC

In this paper, the shape of the stress–strain curves for jujube nucleus concrete (JNC) is changed by the addition of jujube nucleus (JN). To tackle the phenomenon, a constitutive model of JNC corresponding to the requirement of structure design is needed, which is important for both practical engineering design and numerical calculations. 

As shown in Figure 6 and Figure 8, the stress–strain curves of JNC present a longer linear stage than ordinary concrete with the rise in the JN replacement ratio and the elevated temperature and show more obvious elastic characteristics, which is typical for lightweight aggregate concrete. At present, large amounts of constitutive models concerning lightweight aggregate concrete have been researched. Therefore, based on the experimental test presented herein, a two-stage model adapted from Ding’s model [37] is used to describe the stress–strain relationship of JNC by considering various JN replacement ratios and elevated temperatures. A two-stage model (see Equation (11)) is defined by five parameters: the peak stress of concrete, $f_{c}$, the peak strain of concrete, $\varepsilon_{c}$, the parameter of ascending branch, *A*, *B*, and the parameter of descending branch, *α*_c_. The peak stress of concrete, $f_{c}$, can be calculated by Equations (1)–(3). The peak strain of concrete, $\varepsilon_{c}$, can be expressed by Equation (10). Other parameters (ascending branch parameters, *A* and *B*, descending branch parameter, *α*) need modification by considering the effect of replacement ratio and elevated temperature.

(11) $$\sigma =\left\{ \begin{array}{cc} f_{c}^{}\frac{A(\varepsilon /\varepsilon_{c}^{})+(B-1)(\varepsilon /\varepsilon_{c}^{})}{1+(A-2)(\varepsilon /\varepsilon_{c}^{})+B{\left(\varepsilon /\varepsilon_{c}^{}\right)}^{2}} & 0\leq \frac{\varepsilon }{\varepsilon_{c}^{}}\leq 1\\ f_{c}^{}\frac{\varepsilon /\varepsilon_{c}^{}}{\varepsilon /\varepsilon_{c}^{}+\alpha_{c}{\left((\varepsilon /\varepsilon_{c}^{})-1\right)}^{2}} & \frac{\varepsilon }{\varepsilon_{c}^{}}>1 \end{array}\right.$$

(12) $$A=1.64\times 10^{-3}\;\rho f_{c}^{-1/6}$$

(13) $$B=1.68{\left(A-1\right)}^{2}$$

(14) $$\alpha_{c}=3.67\times 10^{-5}f_{c}^{3}$$

### 4.2. Analysis of Model Parameters

The values of parameters *A*, *B*, and *α*_c_ can be determined through a nonlinear curve fitting for the stress–strain curves of jujube nucleus concrete (JNC) under the effect of the jujube nucleus (JN) replacement ratio and elevated temperature. Specific data are tabulated in Table 6.

#### 4.2.1. Ascending Branch Parameter A

**Figure 13:**
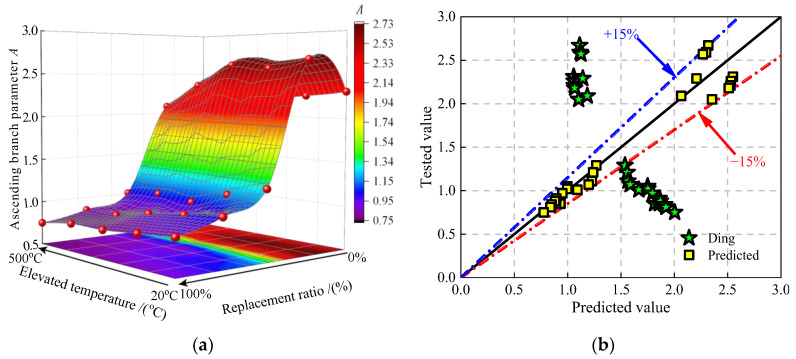
The values of ascending branch parameter *A* for JNC. (**a**) Surface of *A* under the effect of replacement ratios and elevated temperatures. (**b**) Comparison of the tested values and the predicted values [37].

The definition of parameter *A* is the ratio of elastic modulus to peak secant modulus for concrete. Ding et al. [37] shows that the larger the value of parameter *A* is, the longer the linear stage in the ascending branch of stress–strain curve is, and the more the accumulated elastic strain energy of the lightweight aggregate is. Figure 11 shows the values of ascending branch parameter *A* under the effect of the JN replacement ratio and the elevated temperature. It can be seen from Figure 13a that the rise in the JN replacement ratio and the elevated temperature would make the values of the parameter *A* climb to peak when the JN replacement ratio is no more than 25%. It can be concluded that the jujube nucleus has more elastic strain energy under the uniaxial compressive loading than natural aggregate. However, the further rise in the JN replacement ratio and the elevated temperature increases the sucrose molecule content in the JNC, and the values of the parameter *A* decrease rapidly.

Ding et al. [37] shows that parameter *A* can be expressed by the peak stress. However, the predicted values of parameter *A* are discrete when using Equation (12), as shown in Figure 13b. Therefore, Equation (15) is proposed based on the test results for predicting the values of parameter *A*. Figure 13b shows the comparison of the tested values and the predicted values by using Equation (15). It can be seen that the discrepancy of the formula calculation is in the range of ±15%.

(15) $$A=8.95\times 10^{-4}\rho f_{c}^{0.31}$$

where *ρ* stands for the density of the jujube nucleus.

#### 4.2.2. Ascending Branch Parameter B

**Figure 14:**
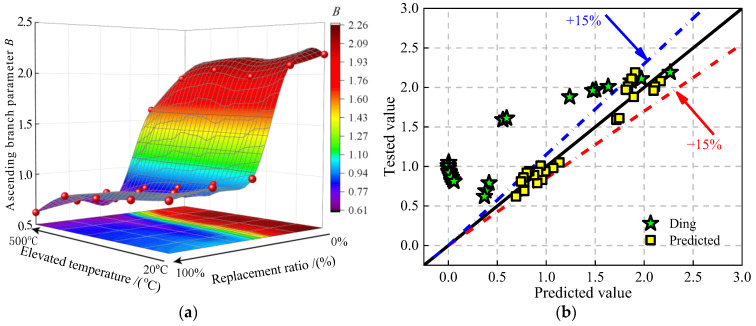
The values of ascending branch parameter *B* for JNC. (**a**) Surface of *B* under the effect of replacement ratios and the elevated temperature. (**b**) Comparison of the tested values and the predicted values [37].

Parameter *B* reflects the degradation degree of the elastic modulus for concrete [37]. Figure 14a presents the values of ascending branch parameter *B* under the effect of JN replacement and elevated temperature. It can be seen that the values of parameter *B* slip down gradually to the bottom with the rise in the JN replacement ratio and the elevated temperature. The difference is that the values of parameter *B* decrease rapidly when the JN replacement ratio exceeds 25%, for the same reason for the decline of the values of parameter *A.*

Ding et al. [37] shows that parameter *B* can be expressed by parameter *A*. However, the predicted values of parameter *B* are overestimated by using Equation (13), as shown in Figure 14b. Therefore, Equation (16) is proposed based on test results to predict the values of parameter *B*. Figure 14b shows the comparison of the predicted values and the test values by using Equation (16). It can be seen that the discrepancy of the formula calculation is in the range of ±15%.

(16) $$B=0.15{\left(A+1.51\right)}^{1.90}$$

where *A* is obtained from Equation (15).

#### 4.2.3. Descending Branch Parameters α_c_

**Figure 15:**
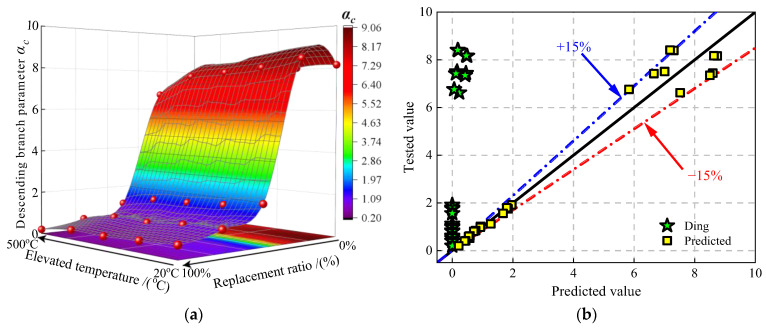
The values of descending branch parameter *α*_c_ for JNC. (**a**) Surface of *α*_c_ under the effect of replacement ratios and elevated temperatures. (**b**) Comparison of the tested values and the predicted values [37].

Parameter *α*_c_ reflects the slope of descending branch curve for concrete [37]. This means that the larger the value of parameter *α*_c_ is, the more obvious the brittle characteristic is. Figure 15a presents the values of descending branch parameter *α*_c_ under the effect of JN replacement ratio and elevated temperature. It can be seen that analogous to parameter *A*, the rise in the JN replacement ratio and elevated temperature would make the values of parameter *α*_c_ climb to the peak when the JN replacement ratio is no more than 25%, which may be because the jujube nucleus has lower stiffness than natural aggregate [31]. When the JN replacement ratio exceeds 25%, the values of parameter *α*_c_ slips down to the bottom rapidly, which is attributed to the same reason for the parameter *A* deterioration.

Similar to parameter *A*, descending branch parameter *α*_c_ can also be expressed by the cubic compressive strength [37]. However, the values of parameter *α*_c_ are overestimated by using Equation (14), as shown in Figure 15b. Therefore, Equation (17) is proposed based on test results to predict the values of parameter *B*. Figure 15b shows the comparison of the predicted values and the test values by using Equation (17). It can be seen that the discrepancy of the formula calculation is in the range of ±15%.

(17) $$\alpha_{c}=\left\{ \begin{array}{cc} 2.13\;f_{c}^{0.51}-1.19 & r\leq 25\%\\ 0.72f_{c}^{0.83} & r>25\% \end{array}\right.$$

### 4.3. Model Validation

**Figure 16:**
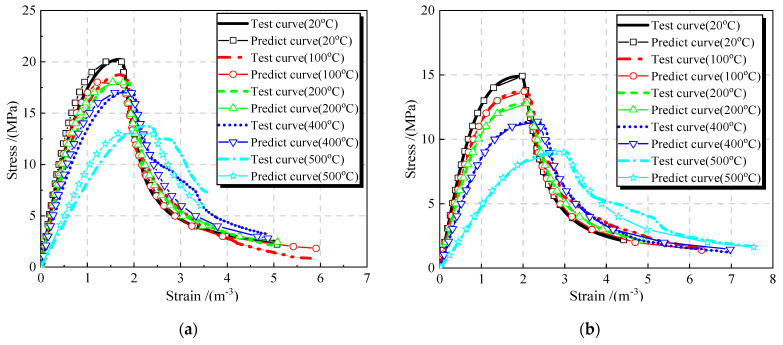

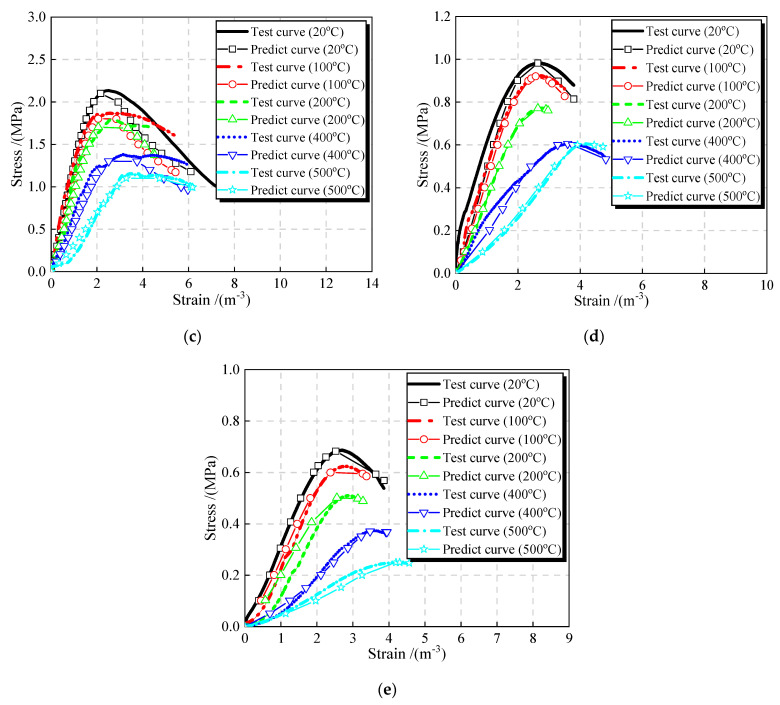
Model validation. (**a**) *r* = 0%. (**b**) *r* = 25%. (**c**) *r* = 50%. (**d**) *r* = 75%. (**e**) *r* = 100%.

As shown in Figure 16, the predicted curves of JNC by using the proposed two-stage model (see Equation (11)) match well with the tested curves of JNC under the effect of the JN replacement ratio and the elevated temperature. Consequently, the proposed two-stage constitutive model herein can be adopted to perform accurate numerical calculation in the future study.

## 5. Conclusions and Next Steps in the Research

This paper investigates the feasibility of using jujube nucleus as a substitute for coarse aggregate in green lightweight aggregate concrete, aiming to provide a solution for natural resources consumption and agricultural waste pollution. The uniaxial compressive behavior of jujube nucleus concrete (JNC) was tested and discussed in detail by considering the effect of the jujube nucleus (JN) replacement ratio and the elevated temperature.

(1) Different from ordinary concrete, the increase in the JN replacement ratio and the elevated temperature would result in the growing number and width of the vertical and diagonal cracks for JNC whilst the linear proportion in the ascending branch and the descending slope increased gradually for the stress–strain curves of JNC, probably due to the low stiffness and high porosity of the jujube nucleus.
(2) The rise in the JN replacement ratio and elevated temperature would decrease the peak stress and elastic modulus but would cause a rapid increase in the peak strain in the stress–strain curves of JNC, which is ascribed possibly to the fact that sucrose molecules can hinder the growth of calcium silicate hydrate and calcium hydroxide to a larger content.
(3) When the JN replacement ratio reaches 25%, the reduction in the peak stress and elastic modulus is 26.36% and 11.27%, respectively, whilst the reduction in the peak stress and elastic modulus of JNC under 100% replacement ratio is 96.60% and 59.48%, respectively. The peak strain of JNC under 25% and 100% replacement ratio increases by 15.93% and 67.24%, respectively.
(4) When the elevated temperature is up to 100 °C, the peak stress and elastic modulus for JNC under 25% replacement ratio decrease by approximately 8.00% and 4.16%, respectively, whilst the reduction increases to 33.00% and 20.52%, respectively, under 400 °C heating. The peak strain of JNC with 25% replacement ratio increases from 2.53% under 100 °C to 19.70% under 400 °C.
(5) A material constitutive model is developed based on the test results for describing the stress–strain relationship of JNC under the effect of the JN replacement ratio and the elevated temperature. The predicted stress–strain curves using the model can match well with the tested stress–strain curves under the different JN replacement ratios and elevated temperatures.

It must be mentioned that the mechanical properties of JNC are acceptable with a 25% substitution ratio, indicating the potential utilization of JNC in pervious, sound-proof, and insolation members towards eco-friendly design. However, the mechanical properties of JNC decrease rather too significantly under a substitution ratio larger than 50% to be of practical interest. Thus, it is necessary to continue the experimental research to refine the formulas for the compressive performance and the stress–strain model of JNC, under substitution ratios between 0% and 35% at small intervals to have more data in this range of ratios. Because normally the durability of lightweight aggregate concrete is not as good as ordinary concrete [39], more studies regarding the durability of JNC are needed in the future. In addition, some reinforcement methods such as hybrid fiber [40] are also worth investigating to improve the mechanical properties of JNC under large substitution ratios. 

## Figures and Tables

**Table 1:** Physical properties of natural aggregates.

	Gradation/mm	Apparent Density/(kg/m^3^)	Water Absorption/%	Crushing Index/%
Natural gravel	5–25	2660	1.4	16.9
Silica sand	0.25–2.5	2580	5.6	7.8

**Table 2:** Physical properties of jujube nucleus.

	Gradation (mm)	Apparent Density (kg/m^3^)	Bulk Density (kg/m^3^)	Water Absorption (by mass)	Tube Compression Strength (MPa)
Big jujube nuclei	5–31.5	1048.56	447.50	32.50%	0.52
Small jujube nuclei	2.36–5	1098.63	598.50	19.50%	0.92

**Table 3:** Concrete mixture proportions in 1 m^3^.

Mixture Mode *^1^	r *^2^/%	Mixture Proportions /(kg/m^3^)					
		Cement	Water	Sand	Gravel	Big Jujube Nucleus	Small Jujube Nucleus
C30-r0	0	380	185	648	1198	0	0
C30-r25	25	380	185	648	898.50	18.15	102.85
C30-r50	50	380	185	648	599	36.30	205.69
C30-r75	75	380	185	648	299.50	54.45	308.55
C30-r100	100	380	185	648	0	72.59	411.39

*^1^: For the nomenclature, taking ‘C30-r0′ as an example, C30 is ordinary concrete grade; r0 stands for 0% jujube nucleus replacement ratio. *^2^: r stands for jujube nucleus replacement ratio.

**Table 4:** Specimen identification.

*r*	Temperature				
	20 °C	100 °C	200 °C	400 °C	500 °C
C30-r0	P-20-r0	P-100-r0	P-200-r0	P-400-r0	P-500-r0
C30-r25	P-20-r25	P-100-r25	P-200-r25	P-400-r25	P-500-r25
C30-r50	P-20-r50	P-100-r50	P-200-r50	P-400-r50	P-500-r50
C30-r75	P-20-r75	P-100-r75	P-200-r75	P-400-r75	P-500-r75
C30-r100	P-20-r100	P-100-r100	P-200-r100	P-400-r100	P-500-r100

**Table 5:** The residual mechanical properties of JNC.

Properties	Number	Ambient	100 °C	200 °C	400 °C	500 °C
*f*_c_ (Mpa)	C30-r0	20.25	18.75	18.09	17.22	13.77
	C30-r25	15.08	13.52	13.18	12.38	10.02
	C30-r50	3.01	2.87	2.79	2.38	2.16
	C30-r75	0.98	0.92	0.77	0.71	0.67
	C30-r100	0.69	0.62	0.51	0.47	0.45
*E*_c_ (GPa)	C30-r0	19.43	19.11	18.96	17.13	15.61
	C30-r25	17.24	16.52	14.83	14.30	13.70
	C30-r50	10.13	9.72	9.05	9.44	8.79
	C30-r75	8.24	8.23	7.40	7.92	7.13
	C30-r100	7.87	7.42	6.69	6.64	6.39
*ε*_c_ (με)	C30-r0	1708.00	1720.00	1750.00	1920.00	2300.00
	C30-r25	1980.00	2030.00	2090.00	2350.00	2370.00
	C30-r50	2505.87	2542.07	2616.47	3154.00	3321.53
	C30-r75	2698.07	2742.07	2776.73	3553.27	3672.00
	C30-r100	2856.47	2952.67	2965.33	3627.40	3909.00

Note: *f*_c_ is the peak stress of JNC; *E*_c_ is the elastic modulus of JNC. *ε*_c_ is peak strain of JNC.

**Table 6:** Parameters of constitutive equation.

Sort	Parameters	20 °C	100 °C	200 °C	400 °C	500 °C
C30-r0	*A*	2.31	2.26	2.21	2.18	2.05
	*B*	2.19	2.11	2.01	1.97	1.59
	*α*_c_	8.16	8.17	7.44	7.35	6.62
C30-r25	*A*	2.51	2.47	2.33	2.27	2.08
	*B*	2.08	2.01	1.96	1.88	1.61
	*α*_c_	8.39	8.41	7.51	7.42	6.76
C30-r50	*A*	1.29	1.21	1.11	1.07	1.01
	*B*	1.05	0.98	0.94	0.83	0.79
	*α*_c_	1.92	1.75	1.8	1.56	1.32
C30-r75	*A*	1.05	1.02	0.97	0.85	0.85
	*B*	1.01	0.93	0.89	0.81	0.69
	*α*_c_	0.82	0.78	0.68	0.61	0.52
C30-r100	*A*	0.91	0.88	0.84	0.81	0.75
	*B*	0.94	0.92	0.86	0.81	0.62
	*α*_c_	0.62	0.6	0.41	0.40	0.20

## Data Availability

The raw/processed data required to reproduce these findings cannot be shared at this time as the data also forms part of an ongoing study.
